# Clinical validation of the RaDI oropharyngeal dysphagia screening tool in older adults

**DOI:** 10.1590/2317-1782/e20250331en

**Published:** 2026-08-03

**Authors:** Raphael Marinho Carvalho, Rafael Maciel de Andrade Lima, Igor Silva de Castro Lima, Luiza Araújo, Nathália de Oliveira Alves, Hipólito Magalhães, Lidiane Maria de Brito Macedo Ferreira

**Affiliations:** 1 Department of Medicine, Federal University of Rio Grande do Norte (UFRN), Natal, RN, Brazil.; 2 Department of Speech-Language Pathology, Federal University of Rio Grande do Norte (UFRN), Natal, RN, Brazil.; 3 Department of Surgery, Onofre Lopes University Hospital (HUOL), Federal University of Rio Grande do Norte (UFRN), Natal, RN, Brazil.

**Keywords:** Deglutition Disorders, Aged, Data Accuracy, Mass Screening, Reproducibility of Results

## Abstract

**Purpose:**

To identify and verify the accuracy of oropharyngeal dysphagia screening in older patients hospitalized or undergoing outpatient follow-up, and to associate the presence of dysphagia with the main comorbidities in this population.

**Methods:**

This is a cross-sectional study with older patients hospitalized in wards or seeking outpatient consultations at a public referral hospital in Northeastern Brazil. The study applied the Oropharyngeal Dysphagia Screening in Older Adults (RaDI), as well as fiberoptic endoscopic evaluation of swallowing (FEES) in a subgroup of patients.

**Results:**

The instrument identified a significant number of older patients with signs suggestive of oropharyngeal dysphagia and showed a consistent association with the reference examination used and an association with neurological, pulmonary, and cardiological comorbidities.

**Conclusion:**

RaDI is a practical and useful tool for dysphagia screening in older people, showing relevant clinical agreement with FEES and potential for application in hospital and outpatient settings.

## INTRODUCTION

Swallowing, an essential physiological function for nutritional and social performance in humans, is a complex biological mechanism that is constantly changing throughout life, making it an opportune field for the development of scientific studies, especially in relation to the mechanisms involved in healthy aging and its differentiation from pathological conditions^(1)^, such as dysphagia in older patients. The concept of dysphagia can be understood as an alteration in the swallowing of liquid or solid substances in one of its phases, whether oral, pharyngeal, or esophageal, leading to difficulty in the passage of food and, in its most severe manifestation, to the occurrence of aspiration^(2)^. In older people, dysphagia can be suspected when the following warning signs are present: delirium, inadequate number of chews, increased meal time, dysarthria, dysphonia, recurrent pneumonia, atrophy, unexplained weight loss, and so forth^(3)^.

Although natural physiological changes occur in the muscles involved in the swallowing process with age, these changes correspond to presbyphagia. This phenomenon causes a natural slowing of swallowing and rarely causes symptoms^(4)^, so it should not be confused with dysphagia in older adults. This situation is a pathological condition that manifests peculiarly in underlying diseases and requires early investigation, as it can present different signs and symptoms throughout the phases of swallowing. In addition, it can be associated with important clinical and functional consequences in older adults, such as sarcopenia, frailty syndrome, malnutrition, anxiety, and depression^(5-7)^.

Dysphagia is estimated to affect 10% to 33% of older people worldwide and can reach up to 52% in institutionalized older adults with malnutrition^(8)^, especially those suffering from neurological diseases^(3,9-11)^. Regarding presbyphagia, the worldwide occurrence in older people is approximately 31%^(12)^. In Brazil, there is a gap in epidemiological data regarding both adults and older adults^(1)^, which may be associated with an underdiagnosis of dysphagia in the country.

Therefore, the correct assessment and interpretation of the pathological form of feeding difficulty, dysphagia, are necessary and justified by its high occurrence in the older population worldwide, by the potential harm to physical and mental integrity that this condition causes to the affected older person, and the scarcity of national studies addressing the topic.

In this scenario, the encouragement of the use of screening tools for dysphagia gains prominence, due to the low cost and the possibility of ecological analysis of various population groups. Regarding dysphagia screening in older people, the Oropharyngeal Dysphagia Screening in Older Adults (RaDI)^(13)^ stands out, with validation and good applicability for outpatients. The possibility of measuring its accuracy with a highly sensitive method for dysphagia assessment, fiberoptic endoscopic evaluation of swallowing (FEES), together with speech-language-hearing assessment^(8)^, can increase the chances of early diagnosis and appropriate treatment of the patient with dysphagia. Similarly, its use can help in the correct differentiation of pathological conditions from those compatible with physiological aging, avoiding interventions in healthy older people, which lead to unnecessary dietary restrictions and worsened quality of life^(14)^.

Thus, this study aimed to identify and verify the accuracy of oropharyngeal dysphagia screening in older patients hospitalized or under outpatient follow-up, as well as to analyze the association between this condition and the main comorbidities of the individuals evaluated.

## METHODS

This project was a cross-sectional study approved under number 3027515 by the Research Ethics Committee of a Public Referral Hospital in Northeastern Brazil. All participants were informed about the research and authorized the use of data with an informed consent form.

The study included participants aged 60 years and older who were being followed up at a public referral hospital in Northeastern Brazil, either on an outpatient basis or in wards. Patients admitted to the intensive care unit, those diagnosed with neurocognitive diseases, and individuals with esophageal dysphagia were excluded.

Participants were recruited by otolaryngologists, otolaryngology residents, medical students, speech-language-hearing pathologists, and speech-language-hearing students. Initially, 288 eligible older individuals were recruited; this number was defined based on the sample size calculation for a 95% confidence level and a 5% sampling error, using the older population of a state in Northeastern Brazil as a parameter. However, throughout the research, there were four losses due to incomplete data, thus totaling 284 individuals.

All participants underwent a clinical history survey and the application of the RaDI^(14)^. According to this questionnaire, there are nine ordinal items whose response options are used to obtain a total score. Eight questions have three response options: “no” (0 points), “sometimes” (1 point), and “always” (2 points); and one question has two alternatives: “no” (0 points) and “yes” (2 points). The total score corresponds to the sum of the nine items, ranging from 0 to 18 points; scores of 4 points or higher are considered indicative of oropharyngeal dysphagia. The only question with just two answer options is: "Have you lost weight due to difficulty swallowing?". The other eight questions with three answer options are: 1. Do you need to swallow food many times to get it down?; 2. Do you strain to swallow?; 3. Do you feel pain when swallowing?; 4. Do you have throat clearing after swallowing?; 5. Does your voice change after swallowing?; 6. Do you choke after swallowing?; 7. Have you had pneumonia after choking?; 8. Do you feel tired after eating?

Of the 284 participants, 52 also underwent FEES^(15)^, performed by a team of otolaryngologists in conjunction with speech-language-hearing assessment of swallowing to confirm the diagnosis of oropharyngeal dysphagia. FEES is an endoscopic examination that assesses swallowing by visualizing the hypopharynx directly through a flexible nasofibroscope under a video system, after administering food in various consistencies and volumes. Through this examination, it is possible to identify findings that compromise the proper functioning of swallowing, such as altered laryngeal sensitivity, food residue in the larynx, food escape before swallowing, and laryngeal penetration and/or aspiration.

The speech-language-hearing assessment aimed to characterize the functional conditions of the stomatognathic system and the clinical aspects of swallowing, including mobility, tonicity, and strength of lips, tongue, and cheeks; mobility of the soft palate; masticatory pattern; dental status; breathing/speech coordination; maximum phonation time; effective cough; and clinical assessment of direct swallowing with different consistencies.

The clinical assessment of swallowing observed oral control, oral transit time, laryngeal elevation, and clinical signs suggestive of impaired swallowing safety, such as cough, throat clearing, and vocal alteration after swallowing. The findings of the clinical speech-language-hearing assessment were considered in combination with FEES, supporting the diagnostic judgment and classification of oropharyngeal dysphagia by consensus among the evaluators.

This study performed FEES without anesthetics, with a 3.2 mm flexible nasofibroscope from Olympus® and a Storz® video system for recording the exams at 30 fps, following the Santoro standardization^(16)^.

All study patients received food offerings prepared according to levels 7, 4, 2, and 0 of the International Dysphagia Diet Standardisation Initiative (IDDSI)^(17)^, using the food thickener Thicken Up Clear by Nestlé®, food coloring, and industrialized powder juice. Three 5 mL offerings and one 10 mL offering were made for levels 2 and 4; for level zero, three 5 mL offerings, one 10 mL offering, and free sips; and for level 7, one colored water cracker was offered.

For the purposes of this study, the diagnosis of oropharyngeal dysphagia was established from operational criteria based on expert consensus, jointly considering the parameters of efficiency and safety of swallowing observed in FEES and in the speech-language-hearing assessment.

Swallowing efficiency was analyzed by the presence and degree of pharyngeal residue in valleculae and pyriform recesses, classified according to the Yale Pharyngeal Residue Severity Rating Scale (YPR-SRS)^(18)^, whose scores range from 1 (absence of residue) to 5 (severe residue).

Posterior oral escape was also considered in the assessment of swallowing efficiency and carefully analyzed, considering its frequency in older people. The isolated presence of posterior oral escape, especially in mild degrees and without association with significant pharyngeal residues or impairment of swallowing safety, was not considered sufficient for the diagnosis of oropharyngeal dysphagia, in line with studies that demonstrate the need for contextualized interpretation of this finding and its adequate reliability when carefully classified^(19)^.

Swallowing safety was assessed by direct observation of laryngeal penetration and/or laryngotracheal aspiration events during or after swallowing, according to the clinical judgment of the evaluation team, based on the criteria suggested by Rosenbek^(20)^.

The final classification of oropharyngeal dysphagia was carried out by consensus among the evaluators, considering the integration of findings related to swallowing efficiency and safety, based on the literature^(18,20-23)^. For the RaDI accuracy analysis, FEES findings were dichotomized into the presence or absence of oropharyngeal dysphagia.

The final FEES report was prepared jointly, with the signature of the speech-language-hearing pathologist responsible for the evaluation, the resident physician in otolaryngology, and the supervising professor of the specialty, characterizing the examination as an interdisciplinary procedure. This approach is aligned with the understanding that the gold standard for swallowing assessment consists of the integration between clinical assessment, as an initial and contextualizing step, and instrumental assessment, including FEES, the latter being fundamental for diagnostic confirmation and stratification of the severity of swallowing disorders.

In this context, instrumental findings should be interpreted in light of the clinical picture. Moreover, validated scales applied to FEES, such as the YPR-SRS^(18)^ and the Rosenbek Penetration and Aspiration Scale^(20)^, contribute to reducing subjectivity and overcoming the limitations of isolated clinical assessment^(24)^.

The number of examinations was limited due to the scarcity of resources at the service where the study was conducted and the unavailability of participants to return another day to perform them.

The data from the questionnaires answered in Google Forms were converted into an Excel spreadsheet and transferred to the Jamovi® software, from which statistical analyses were performed.

Descriptive analyses of the 284 patients were performed with frequency measures and inferential analysis using Fisher's exact test (for a statistical significance of 0.05).

With the 52 patients who underwent FEES, inferential analyses were performed, and associations were tested using Fisher's exact test (for a statistical significance of 0.05) and the phi coefficient, as well as testing the accuracy of RaDI in relation to FEES through sensitivity and specificity values.

## RESULTS

Considering the screening for dysphagia using RaDI, the study identified an estimated presence of oropharyngeal dysphagia in 22.18% of the sample, with 63 of the 284 participants presenting the condition.

The characterization of the research sample reveals that most participants were female (50.4% of patients), used medication continuously (87.7%), a small percentage used a tracheostomy (3.2%), and a considerable portion were smokers (45.8%). Only 3.2% of the individuals interviewed fed themselves through alternative means.

Their mean age was 71.2 years, but patients with positive screening for dysphagia had a slightly higher average (72.8 years for those with positive screening and 70.8 years for those with negative screening). There was no statistical difference between the groups for this variable, revealing the homogeneity of the sample.

**Figure 1:**
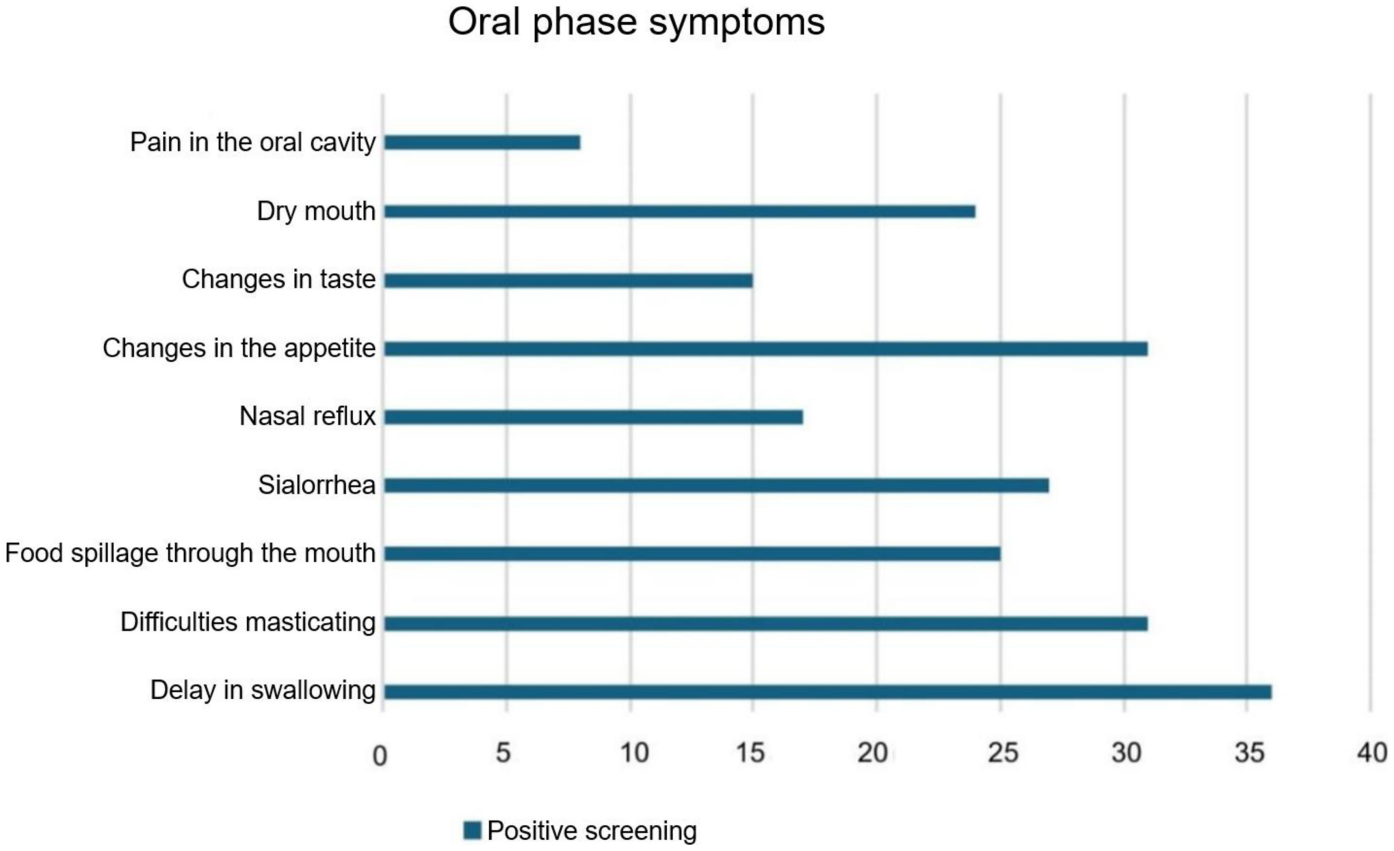
Distribution of patients with dysphagia according to oral phase symptoms

**Figure 2:**
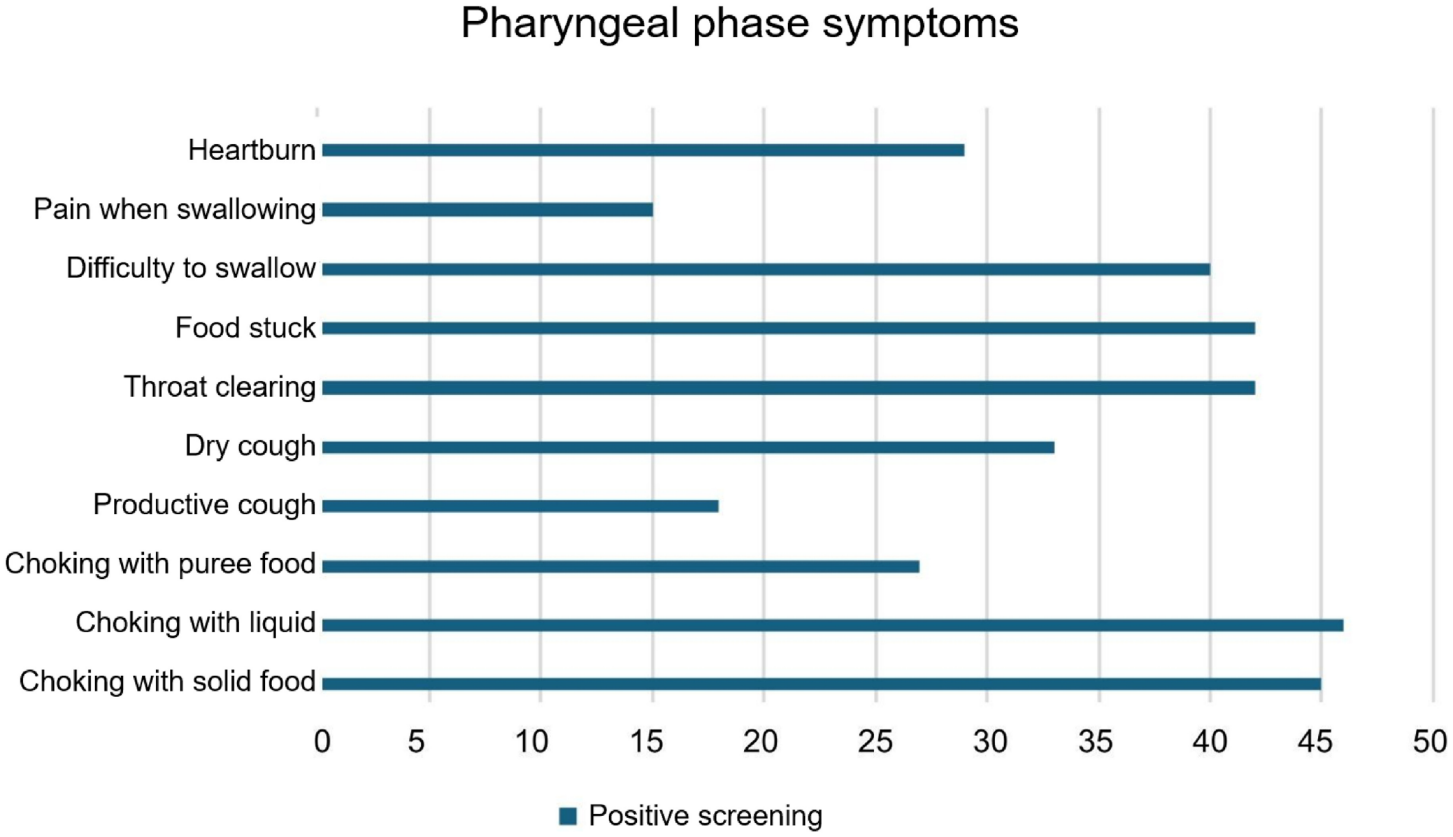
Distribution of patients with dysphagia according to pharyngeal phase symptoms

Regarding the oral phase symptoms reported by patients, the most frequently reported complaints by the group with dysphagia were difficulty swallowing (36 patients), difficulty masticating (31 patients), and changes in appetite (31 patients) (Figure 1). As for the pharyngeal phase symptoms, most individuals with dysphagia reported episodes of choking with solids (45 patients), choking with liquids (46 patients), throat obstruction (42 patients), and throat clearing (42 patients) (Figure 2).

Research in the medical areas involved in dysphagia screening in older people reveals a profile of varied comorbidities. The groups with the highest frequency of findings in patients with dysphagia are those with neurological, gastrointestinal, otolaryngological, endocrinological, oncological, cardiological, and pulmonary comorbidities. From this distribution, we identified a clear association between the presence of neurological, pulmonary, and cardiological diseases and the occurrence of dysphagia (Table 1).

**Table 1:** Associations of comorbidities with dysphagia detected by RaDI

**Comorbidities**	**With dysphagia (RaDI positive)**	**No dysphagia (RaDI negative)**	**Total**	**p**
**Neurological disease**				
Yes	22 (56.41%)	17 (43.58%)	39 (100%)	< 0.0001
No	41 (16.73%)	204 (83.26%)	245 (100%)	
**Gastrointestinal disease**				
Yes	7 (31.81%)	15 (68.18%)	22 (100%)	
No	56 (21.37%)	206 (78.62%)	262 (100%)	0.257
**Otolaryngological disease**				
Yes	7 (21.21%)	26 (78.78%)	33 (100%)	0.886
No	56 (22.31%)	195 (77.68%)	251 (100%)	
**Oncological disease**				
Yes	6 (28.57%)	15 (71.42%)	21 (100%)	0.464
No	57 (21.67%)	206 (78.32%)	263 (100%)	
**Endocrine disease**				
Yes	6 (14.63%)	35 (85.36%)	41 (100%)	0.208
No	57 (23.45%)	186 (76.54%)	243 (100%)	
**Pneumological disease**				
Yes	5 (20%)	20 (80%)	25 (100%)	0.809
No	57 (22.09%)	201 (77.90%)	258 (100%)	
**Heart disease**				
Yes	5 (8.77%)	52 (91.22%)	57 (100%)	0.006
No	58 (25.55%)	169 (74.44%)	227 (100%)	

Fisher's Exact Test

Of the 284 patients evaluated, 52 underwent FEES and speech-language-hearing assessment for dysphagia diagnosis, with the final diagnosis being the presence or absence of dysphagia. Based on this result, the RaDI performance metrics were analyzed in relation to FEES, and accuracy measures were determined using the contingency table (Table 2), finding 75% sensitivity, 75% specificity, and 75% accuracy.

**Table 2:** Performance metrics of the RaDI screening tool based on FEES results

	**FEES: Dysphagia present**	**FEES: Dysphagia absent**	**Total**	**p**	**φ**
**RaDI positive**	*30*	*3*	*33*	*0.004* *	*0.437* **
**RaDI negative**	*10*	*9*	*19*		
**Total**	*40*	*12*	*52*		

* Fisher's Exact Test;

** Phi coefficient

In the analysis of the association between the RaDI variables and the FEES result, Fisher's exact test showed statistical significance (p = 0.004), and the phi coefficient revealed a moderate to strong association (φ = 0.437), demonstrating the clinical relevance of the screening instrument when compared to the gold standard for dysphagia diagnosis, which is FEES associated with speech-language-hearing assessment.

## DISCUSSION

Based on the analysis of the results, a patient profile with a higher probability of oropharyngeal dysphagia was identified: older adult, around 70 years old, using daily medications, and smokers. Knowing this patient profile can guide clinical reasoning and lead to the creation of early monitoring and intervention strategies when there is clinical suspicion of dysphagia. The literature^(25)^ shows that the occurrence and severity of dysphagia increase with age, being more frequent and severe in older people ≥ 85 years, mainly due to degenerative causes, such as presbyphagia and dementia. Among younger people (65-74 years), organic causes stand out, such as head and neck cancer, showing that advancing age influences the type and impact of dysphagia.

The use of tracheostomy and/or alternative feeding routes is already recognized as a risk factor for dysphagia^(9)^. However, only 3.2% of the patients evaluated fell into these contexts, limiting the analysis of these factors.

Oropharyngeal dysphagia was identified in 22.18% of the sample, a finding corroborated by literature data describing dysphagia as a geriatric syndrome affecting approximately 17.3% of the population^(12)^. Given that the population of our study consisted of older people undergoing some type of treatment in the hospital, either outpatient or inpatient, a slightly higher occurrence than that described for the older population in general would be expected. Specific groups, such as older residents of long-term care facilities, may have higher occurrences, with values ​​found in meta-analyses^(26,27)^ between 35.9% and 56.11%, possibly because they are a frailer population.

The most common symptomatology described by the participants surveyed included difficulty swallowing, choking on liquids and solids, a feeling of something stuck in the throat, and throat clearing. Dysfunctions in the pharyngeal phase of swallowing can lead to events such as choking, throat clearing, and coughing, which are manifestations of food penetration or aspiration, representing a significant risk to patient safety and requiring careful clinical evaluation to identify failures in airway closure or bolus propulsion. Oral cavity pain was the least frequent symptom in this study, but it deserves attention because it is bothersome and present in several differential diagnoses.

A relationship was also observed between neurological and cardiological comorbidities and oropharyngeal dysphagia, especially neurological ones, present in more than 50% of affected patients. This pattern is consistent with findings from recent studies^(3,28)^ that showed that the occurrence of oropharyngeal dysphagia is significantly higher among geriatric patients with neurological comorbidities, such as stroke and dementia. Dysphagia, in addition to compromising quality of life, increases the risk of pulmonary aspiration, malnutrition, and repeated hospitalizations, reinforcing the importance of early screening in all older people with neurological conditions. RaDI proved to be an effective tool for detecting oropharyngeal dysphagia in older adults with neurodegenerative diseases, making the assessment more objective and allowing early investigation and intervention.

The analysis of the subgroup of patients undergoing FEES allowed comparison of the findings with RaDI. Among the 40 patients with abnormalities in the examination, 75% were correctly identified by RaDI, while 9 of the 12 patients without abnormalities (75%) were also classified as negative by the instrument. These results indicate that RaDI has good sensitivity and specificity, being able to correctly identify both patients with dysphagia and those without impairment.

Regarding the assessment of dysphagia in public health, where resources and complementary examinations for diagnosis are not always available, a screening instrument with 75% accuracy proves to be of fundamental importance in preventing complications and initiating therapies early for at-risk groups.

## CONCLUSION

The study demonstrated that RaDI is viable for screening oropharyngeal dysphagia in older people, allowing the identification of suspected cases that should be confirmed through clinical swallowing assessment and instrumental evaluation, with good agreement with the FEES and speech-language-hearing assessment. Furthermore, dysphagia in older adults was associated with neurological, pulmonary, and cardiologic comorbidities. Therefore, it is believed that its application in hospital and outpatient settings can favor early diagnosis and timely intervention in dysphagia, especially in patients with multiple comorbidities.
