# Supplementary material for: Clinical validation of the RaDI oropharyngeal dysphagia screening tool in older adults
Source: Codas. 2026 Aug 3;38(5):e20250331. doi: 10.1590/2317-1782/e20250331en (PMC13445990; doi:10.1590/2317-1782/e20250331en)
Supplement: Translation

# Translation: Validação clínica do questionário RaDI para rastreamento de disfagia orofaríngea em idosos

**DOI:** 10.1590/2317-1782/e20250331pt

**Authors:** Raphael Marinho Carvalho, Rafael Maciel de Andrade Lima, Igor Silva de Castro Lima, Luiza Araújo, Nathália de Oliveira Alves, Hipólito Magalhães, Lidiane Maria de Brito Macedo Ferreira

**Affiliations:** 1 Departamento de Medicina, Universidade Federal do Rio Grande Norte - UFRN - Natal (RN), Brasil.; 2 Departamento de Fonoaudiologia, Universidade Federal do Rio Grande do Norte - UFRN - Natal (RN), Brasil.; 3 Departamento de Cirurgia, Hospital Universitário Onofre Lopes - HUOL, Universidade Federal do Rio Grande do Norte - UFRN - Natal (RN), Brasil..

**Keywords:** Transtornos de Deglutição, Idoso, Acurácia dos Dados, Rastreamento, Reprodutibilidade dos Testes

## Abstract

**Objetivo:**

Identificar e verificar a acurácia do rastreamento da disfagia orofaríngea em idosos internados ou em acompanhamento ambulatorial hospitalar, bem como associar a presença de disfagia às principais comorbidades dessa população.

**Método:**

Trata-se de um estudo transversal, com participação de idosos internados nas enfermarias ou que buscavam consultas ambulatoriais de um Hospital Público de Referência na Região Nordeste do Brasil. Foi aplicado o questionário Rastreamento de Disfagia em Idosos (RaDI), além da realização de videoendoscopia da deglutição (VED) em um subgrupo de pacientes.

**Resultados:**

O instrumento identificou um número relevante de idosos com sinais sugestivos de disfagia orofaríngea, além de mostrar associação consistente com o exame de referência utilizado e associação com comorbidades neurológicas, pulmonares e cardiológicas.

**Conclusão:**

O RaDI é uma ferramenta prática e útil para o rastreamento de disfagia em idosos, apresentando concordância clínica relevante com a VED e potencial para aplicação em contextos hospitalares e ambulatoriais.

## INTRODUÇÃO

A deglutição, função fisiológica essencial para desempenho nutricional e social ao ser humano, consiste em um mecanismo biológico complexo e em constante mudança ao longo da vida, tornando-a campo oportuno para o desenvolvimento de estudos científicos, sobretudo em relação aos mecanismos envolvidos em seu envelhecimento saudável e sua diferenciação de acometimentos patológicos^(1)^, tais como a disfagia em pacientes idosos. O conceito de Disfagia pode ser entendido como uma alteração na deglutição de substâncias líquidas ou sólidas em uma de suas fases, seja oral, faríngea ou esofágica, que leva à dificuldade de passagem de alimentos e, em sua manifestação mais grave, à ocorrência de aspiração^(2)^. Em idosos, a disfagia pode ser suspeitada quando há os seguintes sinais de alerta: delirium, inadequado número de mastigações, tempo aumentado da refeição, disartria, disfonia, pneumonias de repetição, atrofia, perda de peso inexplicável, entre outros^(3)^.

Apesar de haver ocorrência de alterações fisiológicas naturais na musculatura envolvida no processo deglutitório com o avançar da idade, essas mudanças correspondem à presbifagia. Este fenômeno ocasiona uma lentificação natural da deglutição e raramente causa sintomas^(4)^, por isso não deve ser confundida com o acometimento do distúrbio da disfagia em idosos. Esta situação é uma condição patológica, que se manifesta de forma peculiar em doenças de base e que demanda investigação precoce, pois pode apresentar diferentes sinais e sintomas ao longo das fases da deglutição. Além disso, pode estar associada, em idosos, a importantes repercussões clínicas e funcionais, tais como sarcopenia, síndrome de fragilidade, desnutrição, ansiedade e depressão^(5-7)^.

No mundo, estima-se que a disfagia acometa entre 10% e 33% dos idosos, podendo chegar até 52% nos idosos institucionalizados com desnutrição^(8)^, especialmente os que sofrem de doenças neurológicas^(3,9-11)^. No tocante à presbifagia, a ocorrência mundial em idosos é de, aproximadamente, 31%^(12)^. No Brasil, há uma lacuna de dados epidemiológicos tanto em relação à população adulta quanto à idosa^(1)^, o que pode estar associado a um subdiagnóstico de disfagia no país.

Dessa forma, a avaliação e interpretação corretas da forma patológica da dificuldade alimentar, a disfagia, são necessárias e justificadas pela sua elevada ocorrência na população idosa mundial, bem como pelo potencial prejuízo à integridade física e mental que esta condição proporciona ao idoso acometido e à escassez de estudos nacionais abordando o tema.

Nesse cenário, o estímulo ao uso de ferramentas de triagem para disfagia ganha notoriedade, devido ao baixo custo e à possibilidade de análise ecológica de vários grupos populacionais. Em relação ao rastreamento de disfagia em idosos, destaca-se o questionário de Rastreamento de Disfagia Orofaríngea em Idosos (RaDI)^(13)^, com validação e boa aplicabilidade para pacientes ambulatoriais. A possibilidade de medir sua acurácia com método de alta sensibilidade para avaliação de disfagia, a Videoendoscopia da Deglutição (VED), juntamente com a avaliação fonoaudiológica^(8)^, pode ampliar as chances de realizar o diagnóstico precoce e tratamento adequado do paciente com disfagia. De maneira semelhante, seu uso pode auxiliar na diferenciação correta de condições patológicas daquelas compatíveis com o envelhecimento fisiológico, evitando intervenções em idosos saudáveis, que levam a restrições alimentares desnecessárias e à piora da qualidade de vida^(14)^.

Diante disso, o presente estudo teve por objetivo identificar e verificar a acurácia do rastreamento de disfagia orofaríngea em idosos internados ou em acompanhamento ambulatorial hospitalar, bem como analisar a associação entre esta condição e as principais comorbidades dos indivíduos avaliados.

## MÉTODO

O presente projeto foi um estudo transversal aprovado sob número 3027515 no Comitê de Ética em Pesquisa (CEP) de um Hospital Público de Referência na Região Nordeste. Todos os participantes foram informados sobre a pesquisa e autorizaram o uso dos dados em vigência do Termo de Consentimento Livre e Esclarecido (TCLE).

Foram incluídos participantes com idade a partir de 60 anos que estavam em acompanhamento em um Hospital Público de Referência na Região Nordeste do Brasil, em regime ambulatorial ou em enfermarias. Foram excluídos pacientes internados em unidade de terapia intensiva, aqueles com diagnóstico de doenças neurocognitivas e indivíduos com disfagia de origem esofágica.

O recrutamento dos participantes foi realizado por médicos otorrinolaringologistas, residentes em otorrinolaringologia, estudantes de medicina, fonoaudiólogos e estudantes de fonoaudiologia. Inicialmente, foram recrutados 288 idosos elegíveis, sendo este número definido a partir do cálculo amostral para 95% do nível de confiança e 5% de erro amostral, tendo como parâmetro a população de idosos de um estado do nordeste do Brasil. Entretanto, ao longo da pesquisa, houve quatro perdas devido ao preenchimento incompleto dos dados, totalizando assim 284 indivíduos.

Todos os participantes foram submetidos a uma anamnese clínica e à aplicação do questionário RaDI (Rastreamento de Disfagia Orofaríngea em idosos - Self-reported Oropharyngeal Dysphagia Screening for Older Adults)^(14)^. Conforme este questionário, há nove itens ordinais cujas opções de resposta são utilizadas para obter um escore total. Oito questões possuem três opções de resposta [“não” (0 ponto), “às vezes” (1 ponto) e “sempre” (2 pontos)], e uma questão possui duas alternativas: “não” (0 ponto) e “sim” (2 pontos). O escore total corresponde à soma dos nove itens, variando de 0 a 18 pontos, sendo considerado indicativo de disfagia orofaríngea a partir de quatro pontos. A única pergunta com apenas duas opções de resposta é: “Perdeu peso por ter dificuldade de engolir?”. As demais oito questões com três opções de resposta são: 1. Precisa engolir muitas vezes o alimento para fazê-lo descer?; 2. Faz esforço para engolir?; 3. Sente dor ao engolir?; 4. Tem pigarro depois de engolir?; 5. Sua voz modifica depois de engolir?; 6. Tem engasgo depois de engolir?; 7. Teve pneumonia depois de algum engasgo?; 8. Sente cansaço depois de comer?

Dos 284 participantes, 52 foram submetidos também à VED^(15)^ realizada por equipe de otorrinolaringologistas em conjunto com avaliação fonoaudiológica da deglutição, com o intuito de confirmar o diagnóstico de disfagia orofaríngea. A VED consiste em um exame endoscópico, no qual a deglutição é avaliada sob visão direta na hipofaringe através do nasofibroscópio flexível sob sistema de vídeo, com administração de alimentos em diversas consistências e volumes. Através deste exame, é possível identificar achados que comprometem o bom funcionamento da deglutição, tais como alteração de sensibilidade laríngea, presença de resíduo alimentar na laringe, presença de escape do alimento antes da deglutição, presença de penetração e/ou aspiração laríngeas.

A avaliação fonoaudiológica teve como objetivo caracterizar as condições funcionais do sistema estomatognático e os aspectos clínicos da deglutição, contemplando: mobilidade, tonicidade e força de lábios, língua e bochechas; mobilidade do palato mole; padrão mastigatório; estado dentário; coordenação pneumofonoarticulatória; tempo máximo de fonação; presença de tosse eficaz; e avaliação clínica da deglutição direta com diferentes consistências.

Durante a avaliação clínica da deglutição, foram observados o controle oral, o tempo de trânsito oral, a elevação laríngea e a presença de sinais clínicos sugestivos de comprometimento da segurança da deglutição, tais como tosse, pigarro ou alteração vocal após a deglutição. Os achados da avaliação fonoaudiológica clínica foram considerados de forma integrada à VED, subsidiando o julgamento diagnóstico e a classificação da disfagia orofaríngea por consenso entre os avaliadores.

No presente estudo, a VED foi realizada sem uso de anestésicos, com nasofibroscópio flexível de 3,2 mm da marca Olympus® e sistema de vídeo Storz® para gravação dos exames a 30fps, além de seguir a padronização de Santoro et al.^(16)^.

Neste estudo, todos os pacientes receberam ofertas de alimentos preparados de acordo com os níveis 7, 4, 2 e 0 do *International Dysphagia Diet Standardisation Initiative (IDDSI)* ^(17)^, para isso utilizou-se o espessante alimentar *Thicken Up Clear*, da marca Nestlé®, corante comestível e suco em pó industrializado. Foram realizadas 3 ofertas de 5mL e 1 oferta de 10mL para os níveis 2 e 4; para o nível zero, 3 ofertas de 5mL, 1 oferta de 10mL e goles livres; e para o nível 7, ofertou-se 1 unidade de biscoito água e sal corado.

Para fins deste estudo, o diagnóstico de disfagia orofaríngea foi estabelecido a partir de critérios operacionais baseados no consenso de especialistas, considerando conjuntamente os parâmetros de eficiência e segurança da deglutição observados na VED e na avaliação fonoaudiológica.

A eficiência da deglutição foi analisada pela presença e pelo grau de resíduos faríngeos em valéculas e recessos piriformes, classificados de acordo com a *Yale Pharyngeal Residue Severity Rating Scale* (YPR-SRS)^(18)^, cujos escores variam de 1 (ausência de resíduo) a 5 (resíduo grave).

O escape oral posterior também foi levado em consideração na avaliação da eficiência da deglutição e analisado de forma criteriosa, considerando sua frequência na população idosa. A presença isolada de escape oral posterior, especialmente em graus leves e sem associação com resíduos faríngeos significativos ou comprometimento da segurança da deglutição, não foi considerada suficiente para o diagnóstico de disfagia orofaríngea, em consonância com estudos que demonstram a necessidade de interpretação contextualizada desse achado e sua adequada confiabilidade quando criteriosamente classificado^(19)^.

A segurança da deglutição foi avaliada pela observação direta de eventos de penetração laríngea e/ou aspiração laringotraqueal durante ou após a deglutição, conforme julgamento clínico da equipe avaliadora, baseando-se nos critérios sugeridos por Rosenbek^(20)^.

A classificação final da disfagia orofaríngea foi realizada por consenso entre os avaliadores, considerando a integração dos achados relacionados à eficiência e à segurança da deglutição, com base na literatura^(18,20-23)^. Para a análise de acurácia do questionário RaDI, os achados da VED foram dicotomizados em presença ou ausência de disfagia orofaríngea.

O laudo final da VED foi elaborado de forma conjunta, com assinatura da fonoaudióloga responsável pela avaliação, da(o) médica(o) residente em otorrinolaringologia e da professora supervisora da especialidade, caracterizando o exame como um procedimento interdisciplinar. Essa abordagem está alinhada ao entendimento de que o padrão de referência para a avaliação da deglutição consiste na integração entre a avaliação clínica, enquanto etapa inicial e contextualizadora, e a avaliação instrumental, incluindo a VED, sendo esta última fundamental para a confirmação diagnóstica e a estratificação da gravidade dos distúrbios da deglutição.

Nesse contexto, os achados instrumentais devem ser interpretados à luz do quadro clínico, e o uso de escalas validadas aplicadas à VED, como a Yale Pharyngeal Residue Severity Rating Scale^(18)^ e Escala de penetração e aspiração de Rosenbek^(20)^, contribui para a redução da subjetividade e para a superação das limitações da avaliação clínica isolada^(24)^.

O número de exames foi limitado devido à escassez de recursos do serviço em que o estudo foi feito, assim como pela indisponibilidade dos participantes em retornar outro dia para a realização do mesmo.

Os dados dos questionários respondidos no *Google Forms* foram convertidos na planilha em *Excel* e transferidos para o *software* Jamovi®, a partir do qual as análises estatísticas foram realizadas.

Foram realizadas análises descritivas dos 284 pacientes avaliados com medidas de frequência e análise inferencial por meio do Teste Exato de Fisher (para uma significância estatística de 0,05).

Com os 52 pacientes que foram submetidos à VED, foram realizadas análises inferenciais e testadas associações através do Teste Exato de Fisher (para uma significância estatística de 0,05) e Coeficiente Phi, assim como testada a acurácia do RaDI em relação à VED através dos valores de sensibilidade e especificidade.

## RESULTADOS

Considerando-se o rastreamento de disfagia a partir do instrumento RaDI, foi identificado no estudo a presença estimada de disfagia orofaríngea em 22,18% da amostra, com 63 dos 284 participantes apresentando a condição.

A caracterização da amostra da pesquisa revela que a maioria dos participantes era do sexo feminino (50,4% dos pacientes), fazia uso de medicamentos de forma contínua (87,7%), um pequeno percentual fazia uso de traqueostomia (3,2%) e uma parcela considerável era tabagista (45,8%). Apenas 3,2% dos indivíduos entrevistados alimentavam-se por via alternativa.

Em relação à distribuição etária, observamos média de 71,2 anos, sendo que os pacientes com rastreamento positivo para disfagia tiveram média ligeiramente mais elevada (72,8 anos para os com rastreamento positivo e 70,8 anos para os com rastreamento negativo). Não houve diferença estatística entre os grupos para essa variável, revelando a homogeneidade da amostra.

Quanto aos sintomas de fase oral reportados pelos pacientes, as queixas mais referidas pelo grupo com disfagia foram: demora para engolir (36 pacientes), dificuldade de mastigação (31 pacientes) e alteração do apetite (31 pacientes) (Figura 1). Já sobre os sintomas da fase faríngea, observou-se que a maioria dos indivíduos com disfagia relatou episódios de engasgo com sólidos (45 pacientes), engasgo com líquidos (46 pacientes), entalo (42 pacientes) e pigarro (42 pacientes) (Figura 2).

**Figura 1:** Distribuição dos pacientes com disfagia segundo os sintomas da fase oral

**Figura 2:** Distribuição dos pacientes com disfagia segundo os sintomas da fase faríngea

A pesquisa das áreas médicas envolvidas no rastreamento da disfagia em idosos evidencia um perfil de comorbidades variadas, sendo os grupos com maior frequência de achados nos pacientes com disfagia os com comorbidades neurológicas, gastrointestinais, otorrinolaringológicas, endocrinológicas, oncológicas, cardiológicas e pulmonares. A partir desta distribuição, identificamos uma clara associação entre a presença de doenças neurológicas, pulmonares e cardiológicas e a ocorrência de disfagia (Tabela 1).

**Tabela 1:** Associações de comorbidades com disfagia detectadas pelo RaDI

**Comorbidades**	**Com disfagia (RaDI positivo)**	**Sem disfagia (RaDI negativo)**	**Total**	**p**
**Doença neurológica**				
Sim	22 (56,41%)	17 (43,58%)	39 (100%)	< 0,0001
Não	41 (16,73%)	204 (83,26%)	245 (100%)	
**Doença gastrointestinal**				
Sim	7 (31,81%)	15 (68,18%)	22 (100%)	0,257
Não	56 (21,37%)	206 (78,62%)	262 (100%)	
**Doença otorrinolaringológica**				
Sim	7 (21,21%)	26 (78,78%)	33 (100%)	0,886
Não	56 (22,31%)	195 (77,68%)	251 (100%)	
**Doença oncológica**				
Sim	6 (28,57%)	15 (71,42%)	21 (100%)	0,464
Não	57 (21,67%)	206 (78,32%)	263 (100%)	
**Doença endocrinológica**				
Sim	6 (14,63%)	35 (85,36%)	41 (100%)	0,208
Não	57 (23,45%)	186 (76,54%)	243 (100%)	
**Doença pneumológica**				
Sim	5 (20%)	20 (80%)	25 (100%)	0,809
Não	57 (22,09%)	201 (77,90%)	258 (100%)	
**Doença cardiológica**				
Sim	5 (8,77%)	52 (91,22%)	57 (100%)	0,006
Não	58 (25,55%)	169 (74,44%)	227 (100%)	

Teste Exato de Fisher

Dos 284 pacientes avaliados, 52 foram submetidos à VED e avaliação fonoaudiológica para diagnóstico de disfagia, tendo como diagnóstico final presença ou ausência de disfagia. A partir deste resultado, foi realizada a análise das métricas de desempenho do RaDI em relação à VED, e pela tabela de contingência (Tabela 2) foram determinadas medidas de acurácia, tendo sido encontrados valores de 75% de sensibilidade, 75% de especificidade e 75% de acurácia.

**Tabela 2:** Métricas de desempenho do instrumento de rastreamento RaDI com base nos resultados da VED

	**VED: Disfagia Presente**	**VED: Disfagia Ausente**	**Total**	**p**	**φ**
**RaDI positivo**	*30*	*3*	*33*	*0,004* ^*^	*0,437* ^**^
**RaDI negativo**	*10*	*9*	*19*		
**Total**	*40*	*12*	*52*		

* Teste Exato de Fisher;

** Coeficiente Phi

Na análise de associação entre as variáveis RaDI e resultado da VED, o teste Exato de Fisher apresentou significância estatística (p = 0,004) e o Coeficiente Phi revelou associação moderada a forte (φ = 0,437), demonstrando a relevância clínica do instrumento de rastreamento, quando comparado ao padrão-ouro do diagnóstico de disfagia que é a VED associada à avaliação fonoaudiológica.

## DISCUSSÃO

A partir da análise dos resultados, identificou-se um perfil de paciente com maior probabilidade de disfagia orofaríngea: idosa, em torno de 70 anos, em uso de medicações diárias e fumante. Conhecer este perfil de pacientes pode direcionar o raciocínio clínico e levar à criação de estratégias de monitoramento e intervenção precoces quando houver suspeita clínica de disfagia. A literatura^(25)^ demonstra que a ocorrência e a gravidade da disfagia aumentam com a idade, sendo mais frequentes e severas em idosos ≥ 85 anos, principalmente por causas degenerativas, como presbifagia e demência. Já entre os mais jovens (65–74 anos), destacam-se causas orgânicas, como câncer de cabeça e pescoço, evidenciando que o avanço da idade influencia o tipo e o impacto da disfagia.

O uso de traqueostomia e/ou de via alternativa para alimentação já é reconhecido como fator de risco para disfagia^(9)^. Entretanto, apenas 3,2% dos pacientes avaliados se enquadraram nesses contextos, limitando a análise desses fatores.

A disfagia orofaríngea foi identificada em 22,18% da amostra, achado corroborado por dados da literatura que descrevem a disfagia como uma síndrome geriátrica que acomete cerca de 17,3% da população^(12)^. Dado que a população do nosso estudo foi composta por idosos que estavam realizando algum tipo de tratamento no hospital, seja ambulatorialmente ou internados, seria esperada uma ocorrência discretamente superior à descrita para a população idosa em geral. Já grupos específicos, como idosos residentes de instituições de longa permanência, podem ter ocorrências mais elevadas, sendo os valores encontrados nas metanálises^(26, 27)^ entre 35,9% e 56,11%, possivelmente por serem uma população mais frágil.

A sintomatologia mais comum descrita pelos idosos pesquisados incluiu demora para engolir, engasgo com líquidos e sólidos, sensação de entalo e pigarro. Disfunções na fase faríngea da deglutição podem levar a eventos como engasgos, pigarro e tosse, que são manifestações de penetração ou aspiração de alimentos, representando um risco significativo para a segurança do paciente e exigindo avaliação clínica criteriosa para identificar falhas no fechamento da via aérea ou na propulsão do bolo. A dor em cavidade oral foi o sintoma menos frequente neste estudo, mas merece atenção por ser incômoda e estar presente em diversos diagnósticos diferenciais.

Observou-se também uma relação entre comorbidades neurológicas e cardiológicas e disfagia orofaríngea, especialmente as neurológicas, presentes em mais de 50% dos pacientes acometidos. Esse padrão é consistente com achados de estudos recentes^(3,28)^ que evidenciaram que a ocorrência de disfagia orofaríngea é significativamente maior entre os pacientes geriátricos que apresentam comorbidades neurológicas, como acidente vascular cerebral e demência. A disfagia, além de comprometer a qualidade de vida, aumenta o risco de aspiração pulmonar, desnutrição e hospitalizações repetidas, reforçando a importância do rastreamento precoce em todos os idosos com condições neurológicas. O RaDI mostrou-se uma ferramenta eficaz para detectar disfagia orofaríngea em idosos com doenças neurodegenerativas, tornando a avaliação mais objetiva e permitindo investigação e intervenção precoces.

A análise do subgrupo de pacientes submetidos à VED permitiu comparar os achados com o instrumento RaDI. Entre os 40 pacientes com alteração no exame, 75% foram corretamente identificados pelo RaDI, enquanto 9 dos 12 pacientes sem alterações (75%) também foram classificados como negativos pelo instrumento. Esses resultados indicam que o RaDI apresenta boa sensibilidade e especificidade, sendo capaz de identificar corretamente tanto pacientes com disfagia quanto aqueles sem comprometimento.

No tocante à avaliação de disfagia no âmbito da saúde pública, em que nem sempre há disponibilidade de recursos e exames complementares para diagnóstico, um instrumento de rastreamento com acurácia de 75% se mostra como de fundamental importância na prevenção de agravos e início precoce de terapias para os grupos de risco.

## CONCLUSÃO

O estudo demonstrou que o questionário RaDI é um instrumento viável para o rastreamento de disfagia orofaríngea em idosos, permitindo identificar casos suspeitos que devem ser confirmados por meio de avaliação clínica da deglutição e instrumental com boa concordância com a VED e avaliação fonoaudiológica. Além disso, observou-se uma associação entre disfagia em idosos e comorbidades neurológicas, pulmonares e cardiológicas. Diante disso, acredita-se que sua aplicação em contextos hospitalares e ambulatoriais pode favorecer o diagnóstico precoce e a intervenção oportuna na disfagia, especialmente em pacientes com múltiplas comorbidades.
